# Structure and piezo-ferroelectricity relationship study of (K_0.5_Na_0.5_)_0.985_La_0.005_NbO_3_ epitaxial films deposited on SrTiO_3_ by sputtering

**DOI:** 10.1038/s41598-017-17767-3

**Published:** 2017-12-18

**Authors:** H´Linh H´Mŏk, E. Martínez-Aguilar, J. J. Gervacio-Arciniega, X. Vendrell, J. M. Siqueiros-Beltrones, O. Raymond-Herrera

**Affiliations:** 1 Posgrado en Física de Materiales, Centro de Investigación Científica y de Educación Superior de Ensenada, Carretera Tijuana-Ensenada No. 3918, Ensenada, Baja California 22860 Mexico; 2 Centro de Nanociencias y Nanotecnología, Universidad Nacional Autónoma de México AP 14, Ensenada, Baja California 22860 Mexico; 3 CONACYT-Facultad de Ciencias Físico-Matemáticas, Benemérita Universidad Autónoma de Puebla, Av. San Claudio y Av. 18 sur, Col. San Manuel Ciudad Universitaria, Puebla, 72570 Mexico; 4 Department of Materials Science and Engineering, The University of Sheffield, Mappin Street, Sheffield, S1 3JD UK

## Abstract

This work demonstrates that the rf-sputtering technique, combined with appropriate heat treatments, is potentially effective to develop new materials and devices based on oxide-interface and strain engineering. We report a study of the structural-physical properties relationship of high crystalline quality, highly oriented and epitaxial thin films of the lead-free (K_0.5_Na_0.5_)_0.985_La_0.005_NbO_3_ (KNNLa) compound which were successfully deposited on Nb-doped SrTiO_3_ substrates, with orientations [100] (NSTO100) and [110] (NSTO110). The crystalline growth and the local ferroelectric and piezoelectric properties were evaluated by piezoresponse force microscopy combined with transmission electron microscopy and texture analysis by X-ray diffraction. Conditioned by the STO surface parameters, in the KNNLa films on NSTO100 coexist a commensurate [001]-tetragonal phase and two incommensurate [010]-monoclinic phases; while on NSTO110 the KNNLa films grew only in an incommensurate [101]-monoclinic phase. Both samples show excellent *out-of-plane* polarization switching patterns consistent with 180° domains walls; while for KNNLa/NSTO100 ferroelectric domains grow with the polarization pointing *down*, for KNNLa/NSTO110 they prefer to grow with the polarization pointing *up*. Comparing with previous reports on epitaxial KNN films, we find our samples to be of very high quality regarding their crystalline growth with highly ordered ferroelectric domains arrangements and, consequently, great potential for domain engineering.

## Introduction

In the last decade, lead-free piezoelectric compounds have received considerable attention as potential substitutes of toxic lead-based piezoelectric materials such as lead zirconate titanate (PZT). (K_x_Na_1−x_)NbO_3_ (KNN), belonging to the *ABO*_3_ perovskite family, is a promising candidate owing to its high Curie temperature (*T*_*C*_ around 400 °C) and excellent piezoelectric properties^1–5^. Similar to PZT, the phase diagram of the KNN system, resulting from the combination of the ferroelectric KNbO_3_ and the antiferroelectric NaNbO_3_ compounds, has temperature independent morphotropic phase boundaries (MPB) in the phase diagram, specially, around x = 0.5 and x = 0.82 where the dielectric constant, electromechanical coupling, piezoelectric coefficients and remanent polarization are maximized due to the coexistence of different crystalline phases^2–13^. However, from the crystallographic point of view, there is plenty of inconsistence in the literature about the KNN structures reported at room temperature around the MPB regions. Although the great majority of the previous works make use of face centered orthorhombic symmetries *Amm2* or *Bmm2* to describe the X-ray diffraction (XRD) patterns of powder and ceramic KNN samples, all of them coincide in the fact that the orthorhombic unit cell is really a double cell where the ABO_3_ perovskite-type primary cell (primitive lattice) has monoclinic *Pm* symmetry^4,5,11,14,15^. The use of *Amm2* or *Bmm2* symmetries and the consideration that the structure of KNN is orthorhombic in the phase diagram are not adequate from the crystallophysics point of view. These orthorhombic models explain the XRD experiment but they cannot explain satisfactorily the physical properties (polarizability, spontaneous polarization, domain structure, piezoelectric response, among others). The reason is that they do not correspond with the temperature dependent phase transitions, characteristic of the ferroelectric perovskite-type compounds, that occur from the monoclinic *Pm*, rhombohedral *R3m* or orthorhombic *Pnma* (or *Pbnm*) distortions at lower temperatures to the tetragonal *P4mm* distortion at intermediate temperatures and to the cubic *Pm* $\bar{3}m$ structure at higher temperatures^11,14,16,17^. As we discuss below, all this takes special significance when KNN thin films are grown on monocrystalline surfaces which induce or constrain the film´s lattice parameters and preferential growth orientations leading to epitaxiallity^18,19^.

Another challenge is to bring the ferroelectric-ferroelectric transition temperature (*T*_F-F_) of (K_0.5_Na_0.5_)NbO_3_ closer to room temperature (RT) from the reported value of 200 °C^2,20^, to take advantage of the better properties of the tetragonal phase, and the coexistence of different phases. In this respect, many works have been reported about the effect of the dopants on the structural and physical properties of KNN; among them, Li doping has been extensively investigated as a representative modifier for this system^5,11,21^. In 2004, Saito *et al*.^2^ reported textured ceramics of Li-, Ta-, and Sb-modified KNN with dielectric constant of ${\varepsilon }_{33}^{T}/{\varepsilon }_{0}=1570$, normalized strain of S_max_/E_max_ = 750 (pm/V), and amazing values of the piezoelectric constant up to *d*_33_ ~ 416 pC/N, comparable to that of soft PZT ceramics. Moreover, Hao *et al*.^20^ showed that the crystalline structure of (Na_0.5_K_0.5_)_1−3x_La_x_Nb_0.95_Ta_0.05_O_3_ ceramics changed from orthorhombic to pseudo-cubic perovskite phase with the increase of La-doping at room temperature.

In the meantime, driven by the miniaturization and integration tendencies^22,23^, great efforts have been made to fabricate high-quality KNN or KNN-based thin films using different growth techniques such as chemical solution deposition (CSD) via sol-gel procedure^3,13,18,19,24–26^, pulsed laser deposition (PLD)^27,28^, and rf-magnetron sputtering^29–31^. Among them, rf-magnetron sputtering is a particularly attractive technique for applications because it allows large area deposition and is commonly used in actual microelectronic manufactures.

The aim of this work, is to study the structure-piezo-ferroelectric properties relationship of La doped KNN (KNNLa) thin films grown by the rf-magnetron sputtering technique using Nb doped SrTiO_3_ (STO) single-crystal substrates with [100] and [110] crystallographic orientations. The ferroelectric and piezoelectric response of the highly oriented and epitaxial films thus obtained was investigated using piezo-force microscopy, and the strong correlation with the structural and morphological characterization is discussed.

## Experimental

A (K_0.5_Na_0.5_)_0.985_La_0.005_NbO_3_ (KNNLa) ceramic target was synthetized by conventional solid-state reaction using K_2_CO_3_ (99%), Na_2_CO_3_ (99.5%), Nb_2_O_5_ (99.9%), and La_2_O_3_ (≥99.98%) powders as raw materials. The mixed and milled powders were calcined at 700 °C for 2 h, and milled again, pressed at 450 MPa (using PVA as binder), and sintered in air at 950 °C for 2 h. The ∼60 nm thick KNNLa thin films, were deposited by reactive rf-magnetron sputtering on Nb doped SrTiO_3_ single-crystal substrates with [100] (NSTO100) and [110] (NSTO110) crystallographic orientations. The parameters for the deposit were: a base pressure of 2.0 × 10^−5^ Torr, 5 cm target–substrate distance, 585 °C substrate temperature, and 175 Watts rf-power. The deposition time was 1 h and a 4:1 argon/oxygen partial pressure totaling 20 mTorr was used. To enhance the crystalline growth, NSTO substrates and KNNLa/NSTO samples were heat treated under an O_2_ atmosphere at the annealing temperature of 585 °C, before and after the deposition processes. The crystallographic structure and orientation was examined by X-ray diffraction (XRD) using a Panalytical X-Pert Pro MRD diffractometer with monochromatic Cu-K_α1_ radiation (1.540598 Å). The cross-section and the crystal structure of the epitaxial film were analyzed using transmission electron microscopy (TEM) with a JEOL JEMF-2010 microscope. The cross-sectional specimens preparation were realized using a JEOL JIB-4500 scanning electron microscope equipped with focused ion beam technique (SEM + FIB) at room temperature. Structural simulation was carried out using the *VESTA* software (version 3.4)^32^. Surface topography, *out-of-plane* and *in-plane* ferroelectric domain structure and domain switching analysis were studied by piezoresponse force microscopy (PFM) using a Park Systems Launch XE7 atomic force microscope (AFM) with an SR865 lock-amplifier by Stanford Research Systems. Platinum top electrodes were deposited by rf-sputtering. The electromechanical resonance response was measured with an Agilent Precision LCR Meter E4980A source coupled to a two arms CPX-VF cryogenic probe station by LakeShore Cryotronics Inc.

## Results and Discussion

In part A of this section, a combination of XRD, TEM, and AFM techniques and structural simulation are used to investigate the crystalline quality of the epitaxial KNNLa thin films grown on both NSTO substrates (NSTO100 and NSTO110) in regards to the crystalline phases, preferential growth orientations, and epitaxial matching; whereas the *in-plane* lattice parameters are determined by crystallography texture analysis. In part B, the ferroelectric and piezoelectric behavior of the highly ordered ferroelectric domains arrangements are studied by PFM analysis focusing on the structural characteristics.

### A. Structural and morphological characterization

#### KNNLa on NSTO100

**Figure 1:**
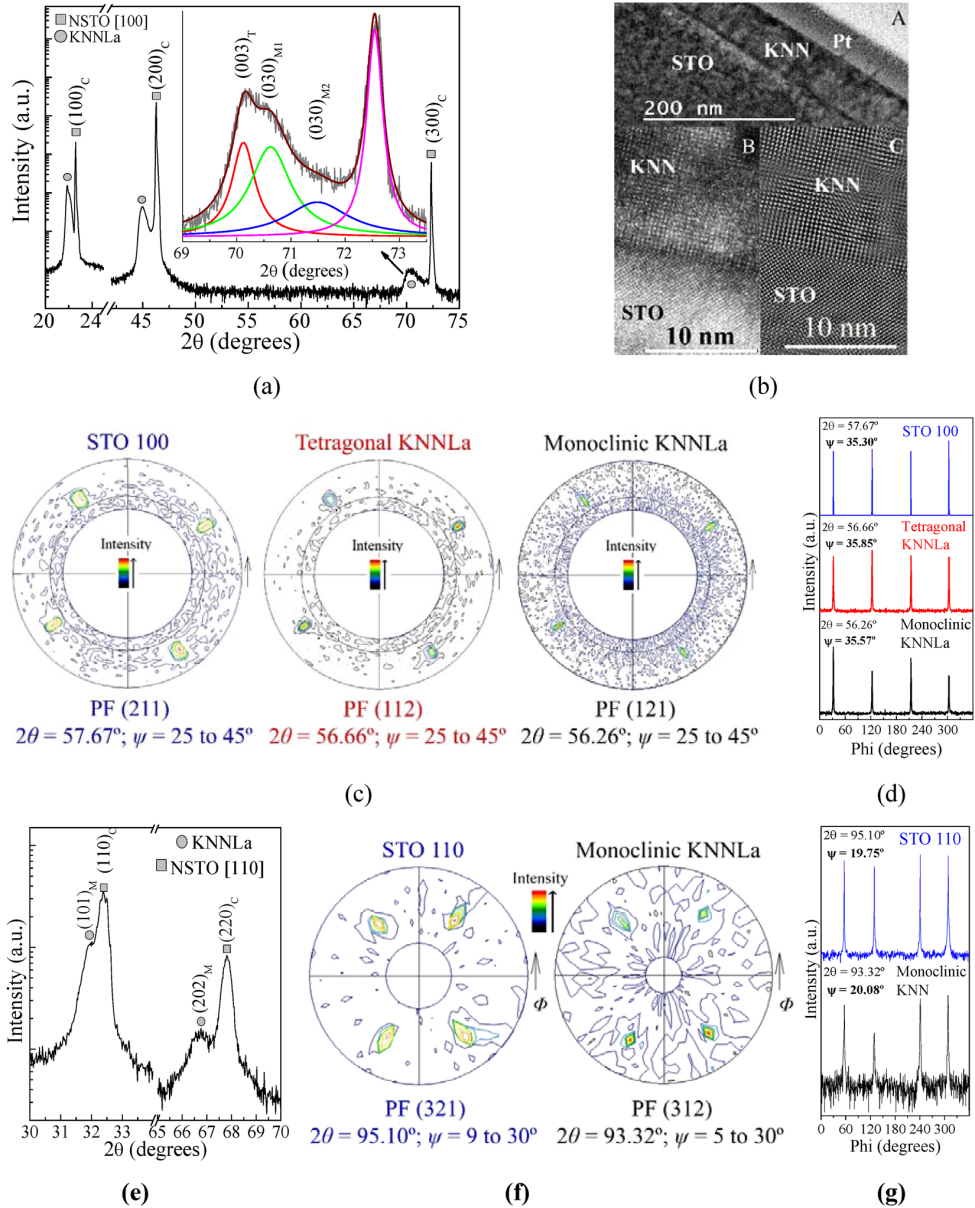
Summary of the structural characterization. (**a**) XRD pattern of the KNNLa film grown on a NSTO100 substrate (K/S1 sample); the inset shows the asymmetry details of the peak near above 2*θ* = 70.20° and the fitting results (4 peaks and the cumulative profile). (**b**) low-resolution (A) and high-resolution (B) TEM cross section images of the K/S1 interface; in (C), the Fourier transform of the interface region in (B). (**c**) (211), (112), and (121) pole figures (PF) obtained for the NSTO100 substrate, and for the tetragonal and monoclinic phases coexisting in the KNNLa film, respectively. (**d**) *Φ*-scans of the PFs in (**c**) at the *ψ*-values corresponding to peak maxima. (**e**) XRD pattern of the KNNLa film grown on a NSTO110 substrate (K/S11 sample). (**f**) (321) and (312) PFs obtained for the NSTO110 substrate and the monoclinic phase in the KNNLa film. (**g**) *Φ*-scans of the PFs in (**f**) at the *ψ*-values corresponding to peak maxima.

Figure 1a shows the XRD pattern of a KNNLa thin film grown on a NSTO100 substrate (labeled K/S1). The strongest peaks correspond to the (100)_C_, (200)_C_, and (300)_C_ planes of the substrate’s cubic perosvkite structure. The peaks at 2*θ* = 21.87°, 45.01° and 70.20°, belong to the pure KNNLa thin film and correspond to a strong *out-of-plane* preferential growth orientation induced by the STO substrate and its *in-plane* lattice parameters at the surface. It is well known from the literature that KNN has a perovskite-like monoclinic structure, stable at room temperature (RT)^3,11,14,33^; however, the asymmetric broadening of the peak at 2*θ* = 70.20° suggests the coexistence of single symmetry phase (monoclinic, orthorhombic, or tetragonal) with different lattice parameters and/or different orientations [such as (*h00*), (*0l0*), and (*00l*)] or more than one phase at RT. The inset of Fig. 1a shows the fitting results, where three peaks centered at 2*θ* = 70.20°, 70.56°, and 71.59° may be deconvoluted and only the third peak at 71.59° is affected by the overlapping with the corresponding peak of the substrate. Consequently, as expected for the (300)_C_ peak (centered at 2*θ* = 72.55°), it is found that the substrate lattice parameter is *a*_STO_ = 3.9060 Å.

**Figure 2:**
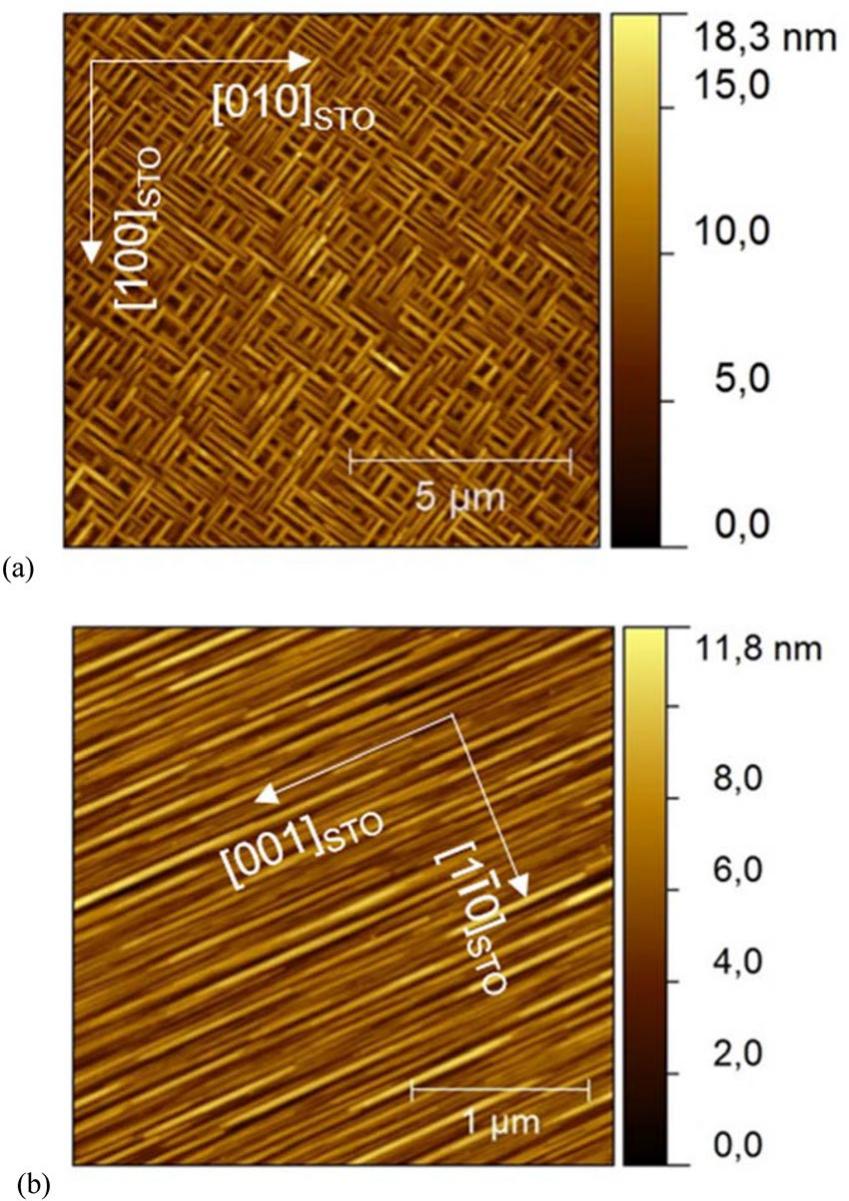
AFM topography images for KNNLa films grown on: (**a**) NSTO100 and (**b**) NSTO110 substrates.

On the other hand, Fig. 2a shows the AFM topography image of the as-grown K/S1 sample. Parallel and perpendicular rod-shape grains with different sizes, forming a maze-like mesh where the rods are oriented at ~45° with respect to the [001] and [010] directions of the STO substrate, are observed. Such arrangement, different to the herringbone domain patterns reported for KNN ceramics^9,12^, may be associated to separated orthogonal, tetragonal or orthorhombic phases, growing locally epitaxial as will be discused below.

To date, similar crystalline growth has not been reported for KNN thin films even for epitaxial growth claimed in different previous works where KNN films were obtained on STO substrates using chemical solution deposition via sol-gel technique^3,19^. In all previous reports, as-grown KNN films show granular morphology with domains distributed randomly *in-plane*, without developing highly ordered crystalline arrangements as observed in Fig. 2.

Moreover, in Fig. 1b, a representative low-resolution TEM cross section micrograph of the K/S1 sample (part A) shows a relaxed KNNLa thin film with an average thickness of 60 nm and a flat and continuous KNNLa/STO interface connection. The high-resolution TEM image (part B) of the KNNLa/STO interface illustrates an epitaxial growth of the KNNLa film following the STO orientation; meanwhile, the Fourier transform of the same region (part C) shows an excellent coupling between the *in-plane* lattice parameters of the KNNLa film and STO substrate with minimal mismatch.

**Figure 3:**
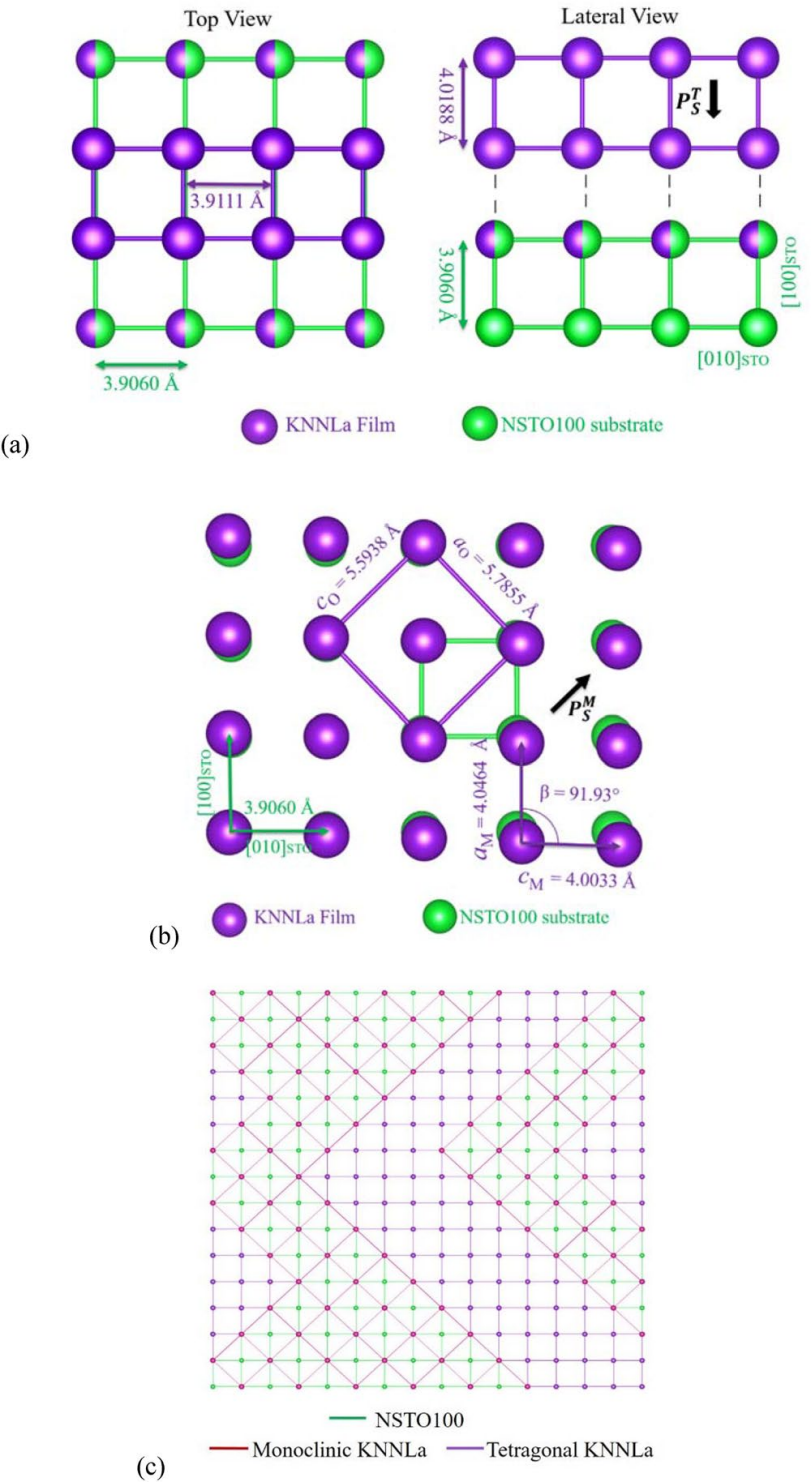
Structural simulation, using the calculated lattice parameters, of: (**a**) commensurate tetragonal (top and lateral views), (**b**) incommensurate monoclinic growth (top view), and (**c**) maze-like pattern (top view) in the KNNLa film grown on the NSTO100 substrate. Only the atoms occupying the A site are illustrated. In (**c**), the face-centered atoms of the orthorhombic cells have been omitted for clearness.

With all those arguments and evidence, and according to the atomic arrangement of the STO’s (100) surface (Fig. 3), the three peaks describing the peak profile at 2*θ* = 70.20° (Fig. 1a) can be associated to the possible coexistence of monoclinic phases found in the vicinity of the MPB or polymorphic regions for x = 0.5^5^ or to the coexistence of such monoclinic phases and a tetragonal one^9,11–13,15^. We consider that the coexistence of tetragonal and monoclinic phases is the best picture to explain the observed results.

Starting with the tetragonal phase, it is assumed to exist at room temperature as result of a strong lattice constriction by compressive stress generated by the STO surface, promoting a strained-epitaxial-like growth; this, in combination with the fact that La doping decreases the transition temperature *T*_F-F_ to around 200 °C^2,20^. However, since for the doped KNN compound the lattice parameters of the reported tetragonal phases are larger than those of the monoclinic phases^4,11,15^, we have assumed here that the peak maxima at 2*θ* = 21.87°, 45.01° and the first deconvoluted peak at 70.20° correspond mainly to the tetragonal contribution. From the XRD pattern, using the 2*θ* values at the maxima of such peaks, where the overlapping is minimum, we can obtain the crystalline parameters with higher precision. Thus, the interplanar spacing for 2*θ* = 70.20°, corresponding to crystalline planes of the KNNLa film parallel to the surface is *d*_1_ = 1.3396 Å. Taking this into account, Fig. 3a shows the model of an epitaxial commensurate growth of a KNNLa film on NSTO100 with tetragonal structure and preferential orientation along [001] assuming that the cell parameters *a*_T_ = *b*_T_ have values close to those of the substrate (3.906 Å) and *c*_T_ = 3*d*_*1*_ = 4.0188 Å obtained from XRD pattern. Therefore, the peaks at 21.87°, 45.01° and 70.20° in the pattern of Fig. 1a can be indexed for the tetragonal phase as (001)_T_, (002)_T_, and (003)_T_, respectively.

As a second possibility, the square array of the STO surface permits to consider an incommensurate growth of the KNN monoclinic primary cell (see Fig. 3b) as has been reported previously^3,19^. In the literature, it has been reported that the monoclinic cell is a special case where two of the parameters are nearly equal (*a*_M_ ≈ *c*_M_) forming an angle β (>90°) and the third one, *b*_M_, is shorter and perpendicular to *a*_M_ and *c*_M_^4,11,14,15^. Therefore, the peaks at 2*θ* = 70.56° and 71.59° (Fig. 1a), corresponding to interplanar spacing of *d*_2_ = 1.3337 Å and *d*_3_ = 1.3170 Å, can be associated with two monoclinic phases M1 and M2 characterized by the parameters *b*_M1_ = 3*d*_2_ = 4.0011 Å and *b*_M2_ = 3*d*_3_ = 3.9510 Å indexed as (030)_M1_ and (030)_M2_, respectively, in agreement with reports by Yu *et al*.^19^.

To obtain further information of the *in-plane* structure (lattice parameters, orientations, and epitaxial quality) of the KNNLa films, crystallography texture analysis was carried out. The *out-of-plane* parameters obtained from XRD patterns and the *in-plane* parameters taken from those reported in the literature for tetragonal and monoclinic phases in correspondence with the STO parameters, were used as starting values. Thus, measurements of pole figures (PFs) for selected (*hkl*) crystalline planes and *ψ*-polar and *Φ*-azimuthal angle scans were realized to obtain the optimal values of 2*θ*_*hkl*_ and *ψ*_*hkl*_. Table 1 summarizes the obtained results for those PFs used to calculate the lattice parameters of each crystalline phase. The analysis for the M2 phase was not considered because of the higher overlapping with the substrate.

**Table 1:** Summary of the texture analysis.

Sample		KNNLa/STO100			KNNLa/STO110	
Phase		Monoclinic	Tetragonal	STO	Monoclinic	STO
Pole Figure parameters		**{130}**	**{103}**	**{310}**	**{302}**	**{320}**
	2*θ*	75.28°	74.82°	77.15°	88.88°	90.67°
	*ψ*	18.25°	18.11°	17.81°	10.88°	10.94°
		**{121}**	**{112}**	**{211}**	**{312}**	**{321}**
	2*θ*	56.26°	56.66°	57.67°	93.32°	95.10°
	*ψ*	35.57°	35.85°	35.30°	20.08°	19.75°
		**{022}**			{$\bar{{\bf{3}}}$ **13}**	
	2*θ*	65.80°			114.94°	
	*ψ*	45.00°			13.5°	
Calculated lattice parameters					*a* = 3.9480 Å	
		*a* = 4.0464 Å			*b* = 3.9114 Å	
		*c* = 4.0033 Å	*a* = 3.9111 Å	*a* = 3.9095 Å	*c* = 4.0197 Å	*a* = 3.9056 Å
		β = 91.93°			β = 90.09°	
					*d*_101_ = 2.8145 Å	
Observed lattice parameters		*b* = 4.0011 Å	*c* = 4.0188 Å	*a* = 3.9060 Å	*d*_101_ = 2.7998 Å	*a* = 3.9058 Å

Pole figures parameters (2*θ* angles and the *ψ* values of the peak maxima), and the lattice parameters calculated from the analysis. The experimental lattice parameters obtained from XRD are presented.

The results demonstrate the coexistence of the tetragonal and monoclinic phases in the KNNLa film of the K/S1 sample. The PFs in Fig. 1c show the expected 4-fold symmetry for the STO and the tetragonal phase present in the KNNLa film. Moreover, due to overlapping between the diffraction peaks of the tetragonal and monoclinic phases, the PF of the tetragonal phase exhibits the presence of the expected 2-fold symmetry of the monoclinic phase, and vice versa, as can be seen from the maxima in the first and third quadrants. Furthermore, the *Φ*-scans in Fig. 1d confirm the strong *in-plane* preferential orientation induced by the STO substrate and the high quality of the epitaxial growth of the KNNLa thin film.

On the other hand, the calculated tetragonal lattice parameters *a*_T_ = *b*_*T*_ = 3.9111 Å are very close to the calculated *a*_STO_ = 3.9095 Å for the substrate which, with the experimental value of *c*_T_ = 4.0188 Å, are in good agreement with the parameters values reported by Sun *et al*.^15^ for 6 mol % Li doped KNN (*a*_T_ = *b*_*T*_ ≈ 3.958 Å and *c*_T_ ≈ 4.025 Å), and by Wang *et al*.^4^ for 8 mol % Li doped KNN (*a*_T_ = *b*_*T*_ ≈ 3.948 Å and *c*_T_ ≈ 4.047 Å). On their behalf, the obtained monoclinic lattice parameters (*a*_M_ = 4.0464 Å, *b*_M_ = 4.0011 Å, *c*_M_ = 4.0033 Å, and β = 91.93°) are in good agreement with the parameter values reported by Tellier *et al*.^14^ (*a*_M_ = 4.0046 Å, *b*_M_ = 3.9446 Å, *c*_M_ = 4.0020 Å, and β = 90.33°) as representative values reported by other authors^10,11^, and with those of the pseudo-cubic parameter of 4.04 Å reported for La-doped KNN thin films obtained by sol-gel by Vendrell *et al*.^34^. Additionally, as can be seen in Fig. 3b, the monoclinic arrangement may be described by means of an orthorhombic cell (SG *Bmm*2) with parameters *a*_O_ = 5.7855 Å and *c*_O_ = 5.6451 Å (computed from the obtained monoclinic values) and *b*_O_ = 4.0011 Å, similar to those reported by Mgbemere *et al*.^35^.

With all this into consideration, the coexistence of tetragonal and monoclinic phases can explain the maze-like topography (Fig. 2a) as is illustrated in the simulation of Fig. 3c. It can also be seen in Fig. 3c, due to the square array of the STO surface, the orthorhombic (monoclinic) growth is possible in the four diagonals forcing the tetragonal growth to follow two directions 90° apart.

#### KNNLa on NSTO110

**Figure 4:**
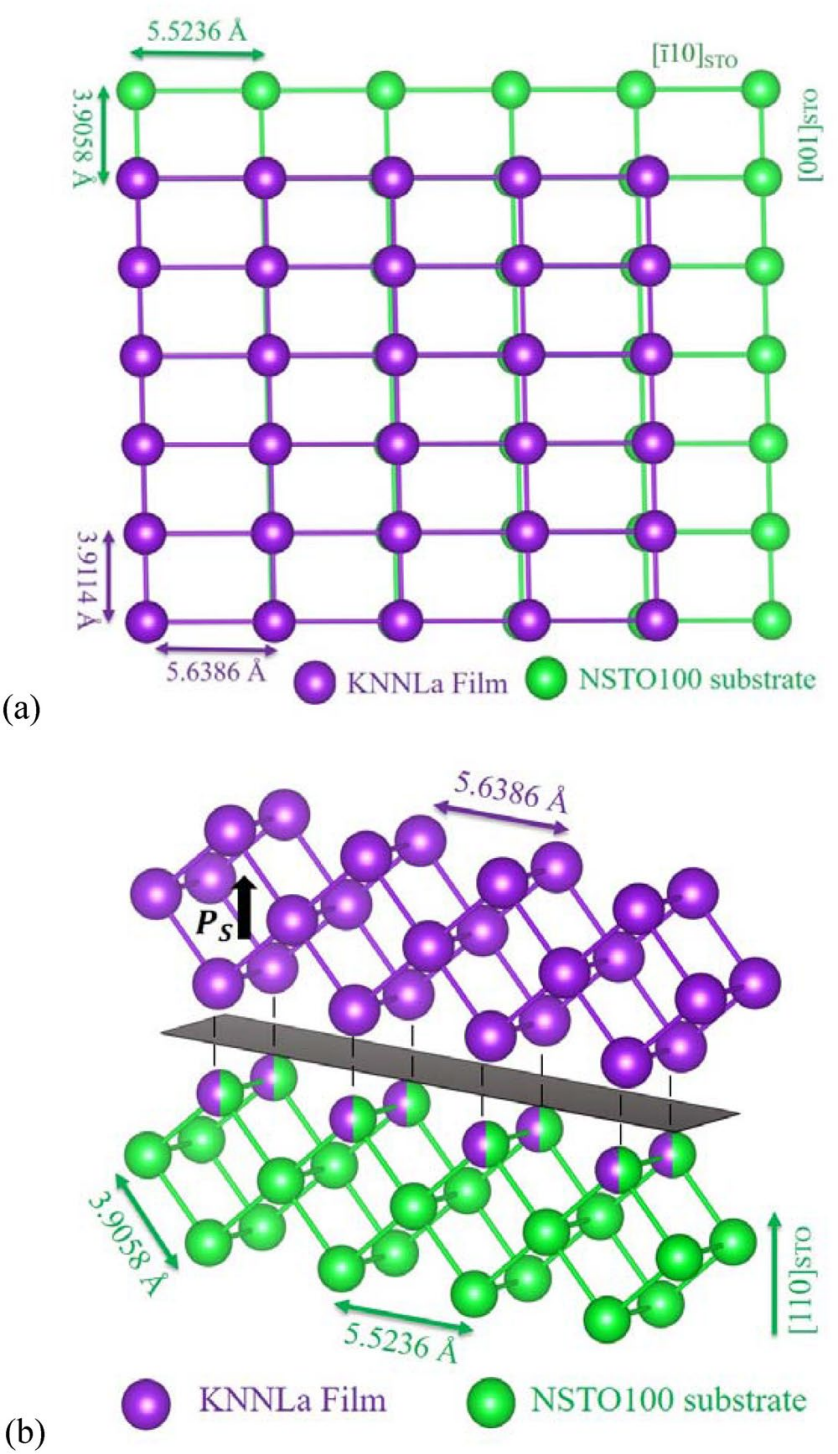
Structural simulation using the calculated lattice parameters of the incommensurate monoclinic growth of the KNNLa film on the NSTO110 substrate. (**a**) Top and (**b**) lateral views. Only the atoms occupying the A site are illustrated.

Figure 1e shows the X-ray diffraction pattern of the KNNLa thin film grown on an NSTO110 substrate (labeled K/S11). The high intensity peaks at *2θ* = 32.49° y *2θ* = 67.81° correspond to the (110)_C_ and (220)_C_ planes of STO, respectively; while, the peaks at *2θ* = 32.01° y *2θ* = 66.77° correspond to the KNNLa film indicating high crystallinity and a strong preferential orientation conditioned by the [110] orientation of the substrate and its *in-plane* lattice parameters at the surface. From the (220)_C_ peak position a value of *a*_STO_ = 3.9058 Å was obtained for the STO in good correspondence with those of NSTO100; thus, the NSTO110 surface is characterized by a rectangular array 3.9058 Å × 5.5236 Å as shown in the simulation in Fig. 4a. Meanwhile, the interplanar spacing obtained from the KNNLa peak at 2*θ* = 66.77° is *d*_4_ = 1.3999 Å. Additionally, Fig. 2b shows the AFM topography image of the as-grown K/S11 sample, where rod-shape grains grow *in-plane* on the substrate surface, mutually parallel along the [001] direction and perpendicular to the [1ī0] directions of the STO substrate.

In this case, we could also think of a (101)-oriented monoclinic phase coexisting with a (110)-oriented tetragonal phase, both of them following the (110) orientation of the substrate as is illustrated in Fig. 4b. Using the parameter value 2*d*_4_ = 2.7998 Å corresponding to planes (101)_M_ or (110)_T_ (consistent with the peak at *2θ* = 32.01°) the value of 3.9595 Å is obtained for the lattice parameters *a*_M_ ≈ *c*_M_ and *a*_T_ = *b*_T_ for the monoclinic and tetragonal phases, respectively. In the light of these results, we can consider that the KNNLa film grows incommensurate for both phases; however, attending to the strong lattice constriction that the NSTO110 surface imposes on the third parameter (*b*_M_ or *c*_T_) to be coupled to its own lattice parameter (*a*_STO_ = 3.9058 Å) by compressive stress, the presence of the tetragonal phase is discarded since the values reported for *c*_T_ are higher than 4.001 Å^4^ as is expected in perovskite like compounds.

Thus, using the experimental value of *d*_101_ = 2.7998 Å and the assumption of *a*_M_ ≈ *c*_M_ = 3.9595 Å as starting model for the monoclinic phase, measurements of PFs for selected (*hkl*) crystalline planes (corresponding to high values of 2*θ* to minimize the overlapping between the diffraction from the KNNLa film and the STO substrate), and *ψ-* and *Φ-*scans were realized to obtain the optimal values of 2*θ*_*hkl*_ and *ψ*_*hkl*_. Table 1 summarizes the obtained results. The PFs in Fig. 1f show the expected 2-fold and mirror (2 *m*) symmetry for the STO and the mirror-symmetry expected for the monoclinic KNNLa film. Meanwhile, the *Φ*-scans in Fig. 1g confirm such symmetries and the strong *in-plane* preferential orientation induced by the STO substrate and the high quality of the epitaxial growth of the KNNLa thin film.

As can be seen in Table 1, the calculated lattice parameter *b*_*M*_ = 3.9114 Å is very close to the calculated *a*_STO_ = 3.9056 Å and the calculated *d*_101_ = 2.8145 Å is consistent with those obtained from the XRD pattern; meanwhile, the rectangular array 3.9114 Å × 5.6386 Å for KNNLa film establishes a mismatch along the [ī01] direction of the STO substrate (Fig. 4a), justifying the assumption made above. Additionally, all computed lattice parameters (*a*_M_ = 3.948 Å, *b*_M_ = 3.9114 Å, *c*_M_ = 4.0197 Å, and β = 90.09°) are in good correspondence with those reported for the monoclinic (orthorhombic) phase of KNN^3,8,36,37^. With this in mind, the peaks in the pattern of Fig. 1e at 2*θ* = 32.01° and 66.77° are indexed as (101)_M_ and (202)_M_, respectively.

### B. Ferroeletric and piezoelectric response

**Figure 5:**
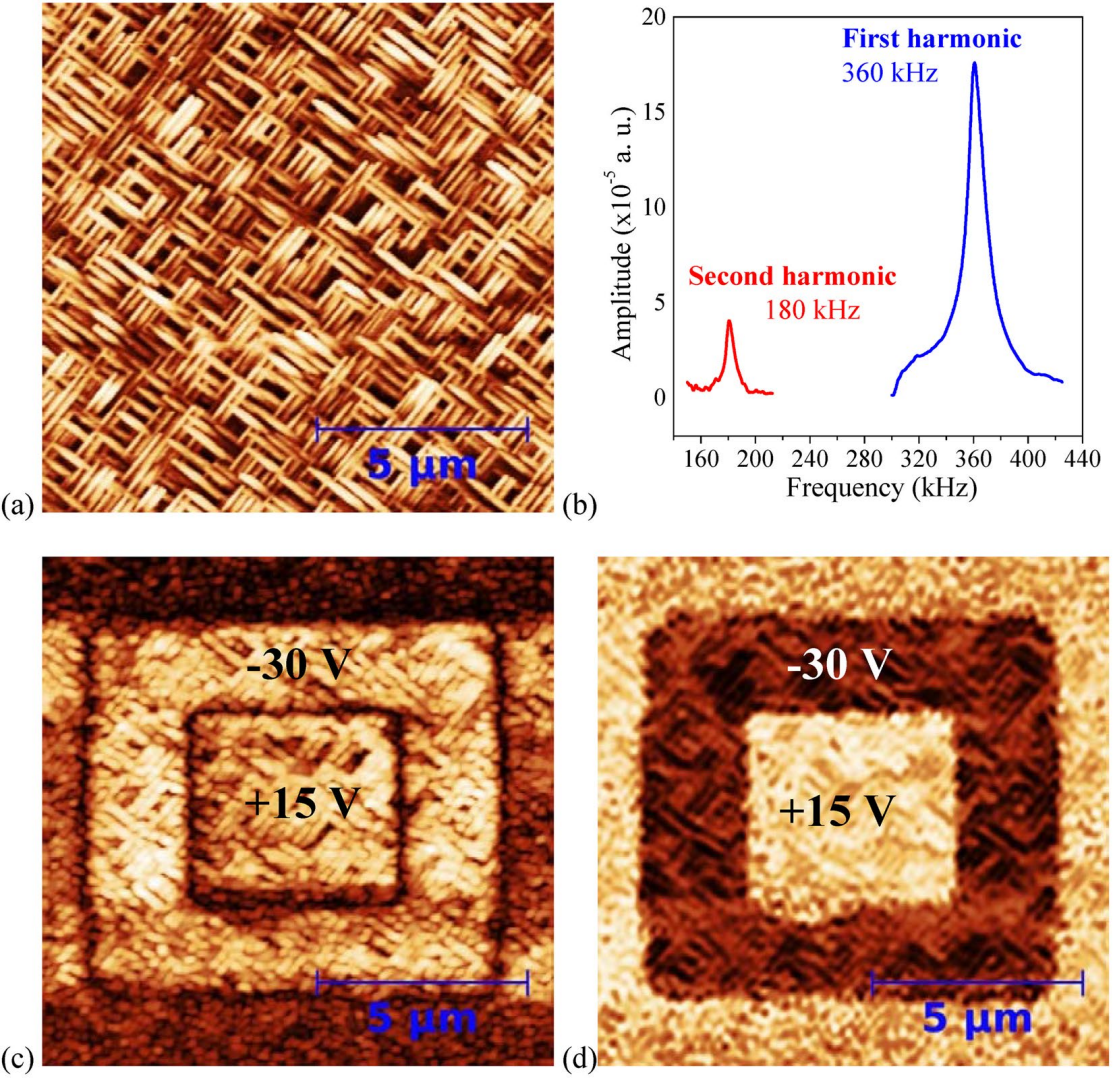
*Out-of-plane* PFM images, for the K/S1 sample, after a poling processes of (**a**) topography, (**c**) amplitude, and (**d**) phase (the bias voltages are indicated). (**b**) First and second harmonic spectra.

**Figure 6:**
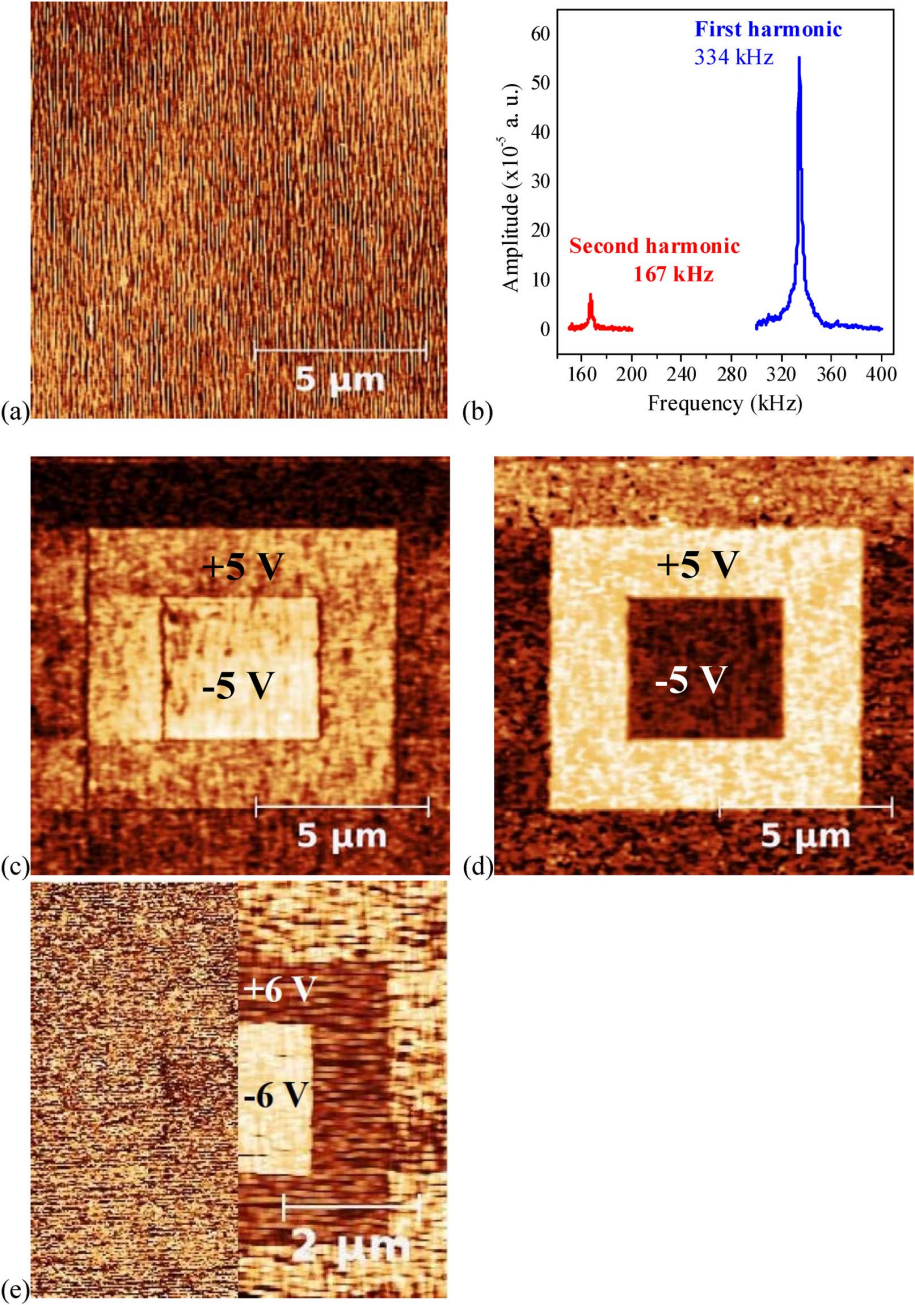
*Out-of-plane* PFM images, for the K/S11 sample, after poling processes of (**a**) topography, (**c**) amplitude, and (**d**) phase (the bias voltages are indicated). (**b**) First and second harmonic spectra. (**e**) *In-plane* (left) and *out-of-plane* (right) phase PFM images, after poling, using a test frequency far away below the first harmonic resonant frequency.

PFM in the resonance mode was used to study the ferroelectric and piezoelectric properties. To investigate the domain structure, the *out-of-plane* polarization switching patterns were explored in a DC regime working at optimal applied voltages on K/S1 (K/S11) sample as follows. In a total area of 12 × 12 µm^2^ of the as-grown sample, an initial poling was carried out by applying a −30 V (+5 V) bias voltage in a concentric 8 × 8 µm^2^ inner area, followed by applying +15 V (−5 V) in a concentric 4 × 4 µm^2^ area enclosed within the previous one. After poling, topography, amplitude, and phase PFM images were simultaneously taken of the full 12 × 12 µm^2^ area. In this work, all measurements were realized with an AC voltage signal of 1 *V*_pp_, at a frequency value near below the maximum resonance in both samples, just where the phase began to change in the electromechanical spectrum. The results are shown in Fig. 5 for K/S1 and Fig. 6 for K/S11. In the topography images of K/S1 (Fig. 5a) and K/S11 (Fig. 6a) no damage was observed as result of the applied electric fields, indicating that the collected PFM signals were coming only from the KNN layers piezoresponse. Moreover, to distinguish if the switching processes, hysteresis loops, and electromechanical properties are originated from the spontaneous polarization, the first/second harmonic criterion was used^38,39^ using AC excitation. In Figs 5b and 6b, the amplitude of the first harmonic is notably higher than that of the second harmonic for both K/S1 and K/S11 samples, respectively, as is expected for good FE materials^39^.

As can be seen in Figs 5c and 6c, the concentric square patterns in the amplitude piezoresponse for both samples show well defined perimeter borders, separating regions with similar expansion and contraction amplitude values, associated to FE domain walls (DW); while, the corresponding concentric patterns in the phase piezoresponse (Figs 5d and 6d) show well defined bright and dark contrast corresponding to *up* and *down* FE domains, respectively; i.e., domains with opposite polarization switched along the *out-of-plane* orientation, characteristic of 180° DWs^9,40^. However, there are some differences between the samples’ behaviors.

Higher domain switching voltages (−30 V and +15 V) were required for the K/S1 sample (Fig. 5) probably due to the coexistence of tetragonal and monoclinic phases where the spontaneous polarization lies along the directions [001] parallel and [101] 90° away from the *out-of-plane* direction, respectively (Fig. 3)^9,12,13,15^. The influence of the coexistence of different crystalline phases on the coercive electric field and the piezoelectric response have been reported for KNN and PZT systems^8,11,16,17,41^. Besides, only half of the voltage (+15 V) was required to reverse the polarization in the innermost area where the phase values are similar to those of the external area, as indication that the FE domains grow with the polarization pointing *down* in good agreement with the preferential growth orientations discussed above.

Meanwhile, lower and symmetric domain switching voltages (+5 V and −5 V) were required for the K/S11 sample (Fig. 6) since only (101)-oriented monoclinic crystallites are aligned parallel to the same *in-plane* substrate direction; where the spontaneous polarization lies along the [101] direction of the monoclinic cell, parallel to the *out-of-plane* direction (Fig. 4b). Moreover, switching is relatively easy for the FE domains grown with the polarization pointing *up* in the external area as can be observed in Fig. 6.

It is worth noting that *in-plane* PFM measurements were realized in both samples on regions as grown, but no contrast was obtained. Figure 6e shows the *in-plane* (left) and *out-of-plane* (right) phase PFM images obtained after poling with switching voltages of +6 V and −6 V applied on concentric square areas of the K/S11 sample. Only the *out-of-plane* phase image shows clearly two areas with opposite polarization, similar to Fig. 6d. In the *in-plane* image no contrast was observed. The piezoresponse signal is very low, compared to those of Fig. 6d, because both measurements can only be simultaneously taken at a test frequency far away below the first harmonic resonant frequency.

Additionally, local polarization hysteresis loops obtained through the phase vs DC voltage curves and local strain loops through the displacement amplitude vs DC voltage curves (butterfly loops) were recorded to obtain quantitative information of the FE domain structure and its electromechanical properties. Such measurements were done with a pulsed triangular DC voltage signal in the ON and OFF field modes; however, as in the ON field mode the loops contain electromechanical and electrostatic effects induced by the capacitive force between the cantilever and the sample surface, only the OFF field mode response was used to minimize such effects^42^. In both samples, a positive bias voltage was applied first.

**Figure 7:**
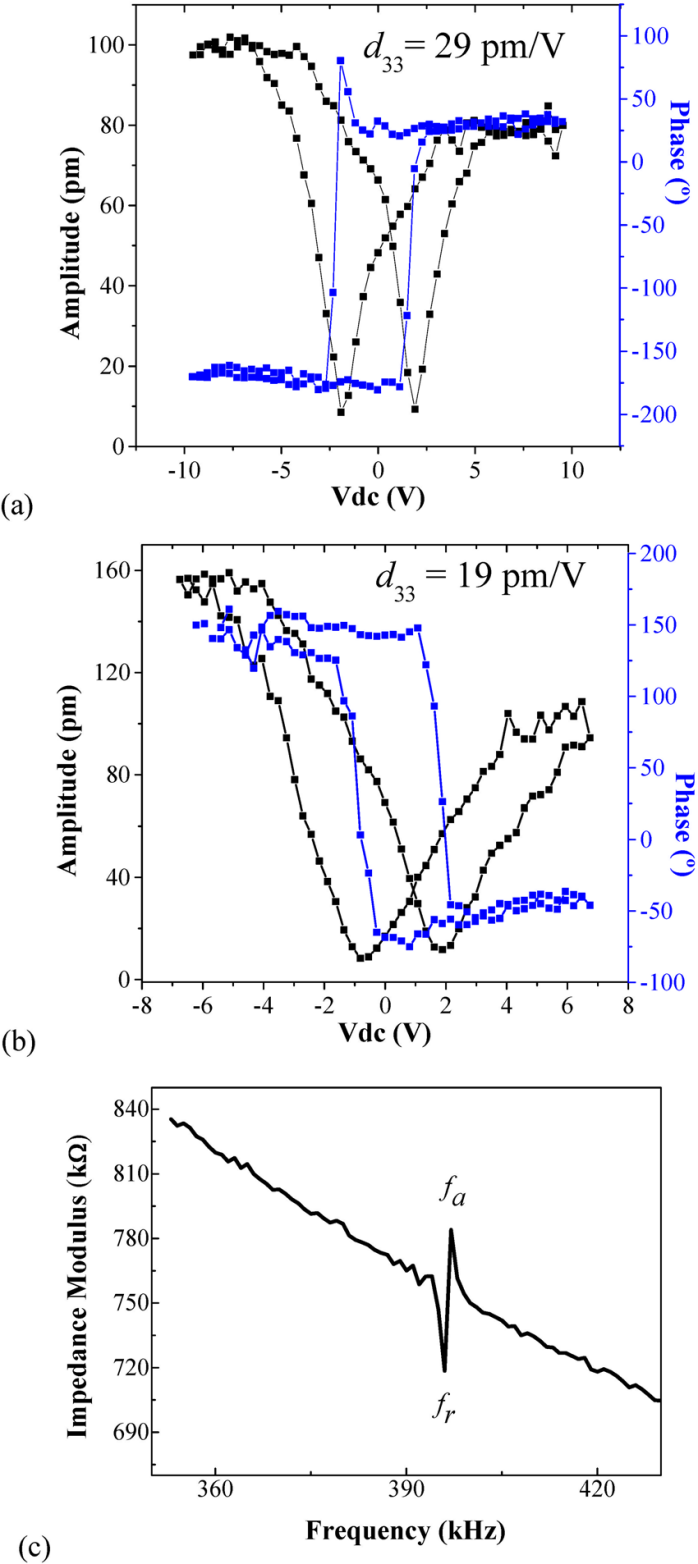
Phase-voltage and amplitude-voltage loops of (**a**) K/S1 and (**b**) K/S11 samples obtained in OFF field mode. (**c**) Impedance modulus spectrum of K/S1 sample.

Figure 7a and b show the phase and butterfly loops for K/S1 and K/S11, respectively. The phase loops are square, typical of 180° DWs, and in both cases the loops are shifted from the origin but in opposite directions, corresponding with the preferential orientation of the polarization, i.e., *down* for K/S1 and *up* for K/S11 as was observed before. Additionally, both samples show low average coercive fields of ~3.7 V for K/S1 and ~2.8 V for K/S11. Meanwhile, according with the phase loops, the samples exhibit asymmetric butterfly loops and the values of the piezoelectric constant *d*_33_ = 29 pm/V for K/S1 and *d*_33_ = 19 pm/V for K/S11 were calculated.

Finally, Fig. 7c shows the global piezoelectric response of the K/S1 sample, obtained through the small-signal impedance modulus spectrum as function of frequency near the electromechanical resonance response. As can be seen, the values of the resonance frequency *f*_*r*_, 396 kHz, and anti-resonance frequency *f*_*a*_, 397 kHz, are in good agreement with the values of the first harmonic in Fig. 5d used in the PFM characterization.

## Conclusions

High quality and highly oriented thin films of the lead-free ferroelectric La-doped (K_0.5_Na_0.5_)NbO_3_ (KNNLa) compound were successfully deposited on SrTiO_3_:Nb[100] and SrTiO_3_:Nb[110] substrates by rf-sputtering. The KNNLa films on [100]-substrate is characterized by the coexistence of a commensurate [001]-oriented tetragonal phase and two incommensurate (010)-oriented monoclinic phases, while on [110]-substrate they grow only in an incommensurate (101)-oriented monoclinic phase, in both cases conditioned by the lattice parameters of the substrates.

The KNNLa films exhibit *out-of-plane* polarization switching patterns of *up* and *down* ferroelectric domains corresponding to 180° domain walls. The as-grown KNNLa films on [100]-substrates show pointing *down* polarization and higher coercivity values depending on the spontaneous polarization direction in each of the coexisting phases. For as-grown KNNLa films on [110]-substrates the domains grow with the polarization pointing *up* with lower coercivity. For both kinds of samples, the obtained piezoelectric constant values are comparable with those in other reports on KNN films. With such ferroelectric and piezoelectric properties, KNNLa films grown by rf-sputtering are potential candidates for applications on ferroelectric lead-free films based devices.
